# Italian Validation of the Feedback Orientation Scale: Psychometric Properties and Cultural Adaptation

**DOI:** 10.3390/bs15121740

**Published:** 2025-12-16

**Authors:** Elena Lo Piccolo, Marco Giovanni Mariani, Gerardo Petruzziello

**Affiliations:** Department of Psychology “Renzo Canestrari”, Alma Mater Studiorum, University of Bologna, 40126 Bologna, Italy

**Keywords:** feedback orientation, scale validation, psychometrics, cross-cultural adaptation

## Abstract

**Background:** Feedback Orientation (FO) reflects how individuals value, accept, and use feedback in a way that influences learning, performance, and sustainable career development. While this concept has been empirically examined, the psychometric properties of the Feedback Orientation Scale (FOS) have received sparse attention outside English-speaking contexts, with no validation in the Italian context. This study aimed to adapt and validate the Italian version of the FOS. **Methods:** A sample of 1092 employees from diverse occupational sectors completed the FOS, also using measures of job satisfaction and perceptions of the feedback environment. The dataset was randomly split to conduct both exploratory and confirmatory factor analyses, followed by reliability and validity testing and measurement invariance analyses across groups. **Results:** Analyses confirmed the original four-factor structure—Utility, Social Awareness, Accountability, and Feedback Self-Efficacy—and supported a higher-order FO construct. The Italian FOS showed acceptable reliability and validity, with expected correlations with job satisfaction and the feedback environment. Measurement invariance was also tested to examine the scale’s equivalence across groups. **Conclusions:** These initial findings provide support for the Italian FOS as a promising instrument with acceptable psychometric properties, extending the cross-cultural understanding of feedback orientation and offering a useful basis for investigating feedback processes in organizational settings.

## 1. Introduction

In today’s rapidly changing work environment, where continuous learning, employability, and sustainability have become essential elements of career development, feedback stands out as a fundamental resource. Far from being a mere evaluative mechanism, feedback has long been recognized as a central factor in organizational learning, employee growth, and performance improvement (Ilgen et al., 1979; Kluger & DeNisi, 1996). It not only informs individuals about their progress toward goals (Tian et al., 2020) but also fosters self-awareness (Martini & Cavenago, 2016), motivation, and the development of transferable skills that strengthen employability (Froehlich et al., 2021)**.** In this sense, feedback plays a pivotal role in promoting individual and organizational adaptability, ultimately contributing to well-being and long-term effectiveness and shaping how individuals cope with the psychological demands of modern work. In particular, the rapid technological change, the constant need for upskilling, and elevated performance expectations have turned feedback into a key mechanism for supporting both psychological resilience and sustainable employability—priorities that align with the United Nations’ 2030 Agenda for Sustainable Development, which sets out the Sustainable Development Goals (SDGs) as a global framework for promoting economic, social, and environmental progress. Among these objectives, SDG 8 (Decent Work and Economic Growth) emphasizes not only the creation of dignified and inclusive working conditions but also the promotion of sustained productivity, skill development, and opportunities for growth. Within this framework, learning-oriented feedback processes become central, as they help individuals acquire new capabilities, improve performance, and adapt to evolving job demands in a sustainable manner.

The long-standing tension between performance management and employee well-being remains a key challenge in organizational settings. As careers become more self-directed and fluid, individuals must navigate continuous change and increased performance demands with limited structural support. In this context, organizations have a responsibility not only to support productivity but also to promote sustainable working lives aligned with the broader priorities of the 2030 Agenda. Equipping the workforce with psychological and behavioral resources—such as feedback receptivity—becomes essential for buffering stress and enabling continuous adaptability. Feedback processes, in particular, serve as both a developmental tool and a practical mechanism for cultivating psychological resilience. As such, they are not merely managerial assets but strategic levers that contribute directly to the creation of decent, inclusive, and sustainable work environments as envisioned in SDG 8.

Among the psychological resources that can support this agenda, Feedback Orientation (FO) has gained increasing attention. Defined as a multidimensional disposition describing how individuals seek, interpret, and use feedback, FO has been linked to more constructive engagement with feedback, enhanced role clarity, and greater adaptability. Importantly, individuals high in FO tend to report lower levels of stress, anxiety, and burnout—highlighting its relevance as a behavioral resource for promoting mental health at work (Dahling et al., 2010; Rasheed et al., 2015; Patel et al., 2019; Katz et al., 2023; Aggarwal et al., 2020). Given its relevance, developing valid and culturally appropriate tools to assess FO is critical for supporting evidence-based HR strategies. In a labor landscape that demands lifelong learning and flexibility, understanding feedback engagement becomes essential not only for performance management but also for safeguarding mental health and sustainable performance and employability.

Individuals, however, differ in how they perceive and use feedback, highlighting the importance of dispositional factors in determining whether feedback is accepted and acted upon (London & Smither, 2002). To address these differences, London and Smither (2002) introduced the construct of Feedback Orientation (FO) conceptualized as a multidimensional disposition that encompasses four dimensions: valuing feedback usefulness (utility), feeling obligated to act on it (accountability), being sensitive to others’ views (social awareness), and having confidence in handling feedback situations (feedback self-efficacy). Building on this framework, Linderbaum and Levy (2010) developed the Feedback Orientation Scale (FOS), integrating London and Smither’s conceptual model with motivational (Vroom, 1964) and attitudinal (Ajzen & Fishbein, 1977) theories. In their initial conceptualization, the authors proposed five dimensions, including *defensiveness*, which they theorized would relate to constructs such as resistance to change. However, empirical analyses demonstrated that defensiveness showed substantial conceptual and statistical overlap with Feedback Self-Efficacy and therefore lacked discriminant validity. Consequently, defensiveness was removed during scale refinement, and resistance to change was considered only as an external correlate in preliminary analyses, not as a core dimension of the FOS.

The validated version of the FOS therefore comprises 20 items capturing four dimensions and a second-order factor derived from London and Smither’s (2002) conceptual model. The scale development process integrated theoretical foundations with both qualitative expert evaluations and quantitative analyses to refine its latent structure. Factor analyses revealed partial conceptual overlaps among some dimensions, leading to a more parsimonious four-factor solution representing distinct yet interrelated aspects of feedback orientation. Subsequent analyses supported a second-order factor structure, indicating that these dimensions reflect manifestations of a single underlying feedback orientation construct.

Despite these advances, empirical studies on the psychometric properties of the *Feedback Orientation Scale* (FOS) remain limited. Since its initial development in the United States (Linderbaum & Levy, 2010), the FOS has been validated in multiple contexts, consistently supporting its four-factor structure and psychometric soundness. In its original validation, the scale showed convergent associations with perceptions of a supportive feedback environment and learning goal orientation. The four subscales demonstrated good internal consistency (α = 0.74–0.86; Linderbaum & Levy, 2010). Criterion-related analyses further demonstrated that FOS scores predict proactive outcomes such as feedback seeking, intentions to use feedback, role clarity, satisfaction with performance appraisal, and participation in developmental activities. Subsequent studies have extended its application to leadership development programs (Braddy et al., 2013), where the overall scale showed high reliability (α = 0.88) and feedback orientation was positively related to incremental implicit person theories, achievement motivation, and positive reactions to 360-degree feedback, though not to external coach ratings. Other validations have confirmed the four-factor structure in specific cultural and professional contexts, including sales professionals in South Africa (Lilford et al., 2014), whose subscales showed excellent internal consistency (α = 0.83–0.92), and health professions students in Chile (Fuentes-Cimma et al., 2025), but with a primary focus on internal factorial validity rather than external correlates. Complementary to this line of research, Kasch et al. (2021) developed the Peer-Feedback Orientation Scale, designed to capture receptivity to peer-delivered feedback across five dimensions (Accountability, Communicativeness, Utility, Feedback Self-efficacy, Receptivity). While the Peer-Feedback Orientation Scale has shown a coherent factorial structure, reliability estimates were reported only at the factorial level; its validation has so far been restricted to internal dimensions, without examining relationships with external constructs. Collectively, these studies demonstrate the robustness of feedback orientation as a construct across cultural and occupational domains, while also underscoring limitations regarding cross-cultural generalizability and the need for further contextual and linguistic adaptations.

While FO has often been treated as a relatively stable individual difference, recent work has described it as a “quasi-trait” that can shift over time in response to sustained feedback experiences (London & Smither, 2002; Katz et al., 2023). Beyond its psychometric validation, research has clarified its nomological network. At the individual level, FO is shaped by mastery goals, age-related patterns (older workers scoring higher on social awareness, younger on utility), motivational tendencies (ego enhancement vs. ego defense), and emotional intelligence, which facilitates constructive feedback use (Leenknecht et al., 2019; Anseel et al., 2013; Wang et al., 2015; Ashford, 2003; Dahling et al., 2010). Contextually, a supportive feedback environment—characterized by trust, openness, and developmental intent—reinforces FO (Dahling et al., 2010).

Consistent with these antecedents, FO predicts positive reactions to feedback, higher job satisfaction, better performance, lower burnout, and greater feedback seeking (Dahling et al., 2010; Katz et al., 2023). It also moderates feedback processes, amplifying the effects of supportive environments on empowerment and fostering creativity by integrating both positive and negative feedback (Gabriel et al., 2014). Overall, FO emerges as a multifaceted construct: shaped by dispositions and context, enhancing feedback reactions and work outcomes, and operating as a boundary condition in feedback processes. As such, it sustains individual learning, well-being, and creativity, while contributing to organizational effectiveness over time.

Building on these premises, the present study adopts a structured validation strategy that goes beyond the examination of internal structure and basic construct validity (e.g., Linderbaum & Levy, 2010; Lilford et al., 2014; Fuentes-Cimma et al., 2025), extending the scope of validation through a more articulated and methodologically robust approach to rigorously assess the Italian version of the FOS. To this end, our objectives are twofold. First, following the methodological recommendations of Worthington and Whittaker (2006) and Brown (2015), we examine the internal validity of the Italian version of the FOS by evaluating its structural validity, internal consistency, and convergent and discriminant validity (Henseler et al., 2015), and by testing its measurement invariance across key socio-demographic groups (Brown, 2015). Second, drawing on the framework proposed by Grimm and Widaman (2012), we investigate its external validity, assessing both criterion-related and nomological validity by examining theoretically grounded associations with relevant external constructs.

By integrating construct validity evidence with an initial examination of measurement invariance, this study provides a balanced and methodologically grounded evaluation of the FOS in the Italian context. These findings offer initial evidence for the reliability and structural validity of the Italian FOS, representing a first validation step in the Italian context. The study provides a preliminary foundation for investigating feedback orientation in Italian workplaces, while highlighting the need for further research to confirm and extend these results.

Providing an empirically supported Italian version of the FOS helps address an existing gap and offers practitioners and researchers a tool for gaining a more nuanced understanding of how employees perceive and use feedback. Although the present validation is not definitive, it supports evidence-informed practices aimed at fostering employee development and may assist practitioners in identifying different levels of feedback receptivity—informing tailored developmental initiatives, leadership pathways, and interventions designed to strengthen feedback-related self-efficacy (Katz et al., 2023). The validated version also establishes a basis for examining whether feedback orientation functions as an antecedent or moderator within feedback processes, and for exploring its links with the broader feedback environment, thereby contributing to the ongoing cross-cultural consolidation of the construct (Fuentes-Cimma et al., 2025).

## 2. Materials and Methods

Prior to conducting quantitative analyses, we carried out a qualitative evaluation of the Italian adaptation of the Feedback Orientation Scale (FOS). The scale was translated and back-translated following standard guidelines (Brislin, 1970) and then reviewed by a panel of subject-matter experts using procedures recommended for qualitative scale refinement (Hennink, 2014; Goetz et al., 2013). During a focus-group session, four work and organizational psychologists jointly examined the Italian and original English items, assessing their semantic clarity, cultural relevance, and potential ambiguity. Items judged redundant, unclear, or conceptually misaligned with the theoretical model were removed at this stage, resulting in a refined 16-item version used for subsequent psychometric analyses (see Appendix A).

This qualitative assessment revealed systematic concerns regarding four items. These items were judged problematic in terms of interpretability, contextual fit, or conceptual clarity, and were therefore removed after the translation and back-translation stage but prior to the exploratory and confirmatory analyses. Specifically, the Utility item “Feedback contributes to my success at work” was considered overly vague, with “success” lacking a consistent operational meaning across sectors. The item Accountability “I feel obligated to make changes based on feedback” raised concerns about multiple possible interpretations—from ethical responsibility to social pressure. The Social Awareness item “I rely on feedback to help me make a good impression” was viewed as redundant and overly centered on impression management. Finally, the Feedback Self-efficacy item “Compared to others, I am more competent at handling feedback” was regarded as misaligned with common evaluative practices, where feedback handling is not typically framed in comparative terms. A synthesis of these qualitative considerations is reported in Table 1, which summarizes the rationale for item removal and supports the adoption of the refined item set used in the subsequent analyses.

This study adopted a cross-sectional design and surveyed employed adults in the Italian labor market via an anonymous online questionnaire, and was designed and reported in accordance with APA JARS–Quant standards for quantitative studies involving new data collection (Appelbaum et al., 2018, Table 1) and Structural Equation Modeling (SEM) reporting guidelines (Appelbaum et al., 2018, Table 7). The research complied with the Guidelines of the Helsinki Declaration and was approved by the Ethics Committee of the authors’ institution (approval code: [0217447]). Participants were recruited using a non-probability convenience and snowball sampling strategy (Etikan, 2016). Data were collected over a period of approximately 10 months, ensuring broad participation across different occupational sectors. The initial recruitment involved undergraduate and graduate students from different degree programs (e.g., psychology, economics, engineering, social sciences) at an Italian university located in Central Italy, who were encouraged to disseminate the survey through their personal networks and social media platforms (e.g., WhatsApp, Instagram, Facebook). This procedure enabled the survey to reach workers employed across different sectors and geographical areas.

To ensure the inclusion of employed individuals, respondents had to meet the following inclusion criteria: (a) being currently employed and (b) working in an organization with at least ten employees, in either the public or the private sector. No incentives were provided for participation.

The final sample comprised 1092 participants (Age = 41.2 years); women were the majority (56.2%), and 0.7% selected “other” or preferred not to disclose gender. Most participants worked in Northern Italy (58.2%), followed by Central (25.5%), Southern and Islands (14.5%), with 1.8% abroad. Although the sample included participants from all major Italian regions, the distribution was not perfectly balanced, with a lower proportion of respondents from Southern Italy. This should be taken into account when considering the generalizability of the results. Employment sectors were diverse, with most representing collective/personal services (15.0%), manufacturing (13.2%), commerce (12.8%), healthcare/assistance (11.2%), and hospitality (11.9%), with additional presence in education (8.2%), banking/insurance (7.1%), and public administration (5.1%). Most participants (76.0%) reported holding non-managerial roles (e.g., employees, clerks, blue-collar workers), while 24.0% reported managerial roles (e.g., functionaries, supervisors, executives). The FOS was translated via back-translation and expert review, following standard guidelines.

The two randomly split subsamples (n = 546 each) were well balanced in terms of socio-demographic composition. This automatic random split ensures that each participant has an equal probability of being allocated to either subsample, thereby reducing selection bias. The use of set.seed(), a function in R to ensure reproducibility of the random split, allows the exact split to be replicated by other researchers, strengthening the methodological transparency of the procedure. In the Exploratory Factor Analyses subsample, 40.8% were male, 58.6% female, and 0.5% identified as other. Educational levels were distributed as follows: 4.2% lower secondary or below, 50.0% high school diploma, 34.6% university degree, and 11.2% postgraduate qualifications. Occupational positions indicated that 75.5% of participants held non-managerial roles (e.g., employees, clerks, blue-collar workers), while 24.5% held managerial roles (e.g., functionaries, supervisors, executives).

The Confirmatory Factor Analyses (CFA) subsample displayed a comparable profile, with 45.2% males, 53.8% females, and 0.9% identifying as other. Educational levels comprised 5.3% lower secondary or below, 50.0% high school diploma, 35.0% university degree, and 9.7% postgraduate qualifications. Similarly, 76.6% of participants reported non-managerial roles, whereas 23.4% reported managerial positions.

### 2.1. Analysis

The following sections report the exploratory and confirmatory analyses conducted on these subsamples, outlining the empirical evaluation of the scale’s dimensionality and measurement quality.

#### 2.1.1. Exploratory Factor Analyses

In the first subsample, the number of factors was determined through parallel analysis, a method shown to outperform traditional criteria such as eigenvalues >1 or scree plots in preventing over- or under-extraction. Consistent with the original validation by Linderbaum and Levy (2010), we conducted an EFA using Principal Axis Factoring (PAF) with oblique rotation (direct oblimin). PAF was selected because it is robust to deviations from multivariate normality, which are common in Likert-type data (Costello & Osborne, 2005), while an oblique solution was appropriate given the theoretical expectation that the four dimensions of feedback orientation would be intercorrelated, reflecting the multidimensional yet related nature of the construct.

#### 2.1.2. Confirmatory Factor Analyses

In the second subsample, Confirmatory Factor Analyses (CFA) were performed to test competing structural models of the FOS derived from theory and prior validations (Linderbaum & Levy, 2010; Braddy et al., 2013; Lilford et al., 2014; Kasch et al., 2021; Fuentes-Cimma et al., 2025). Models included a unidimensional structure, the original second-order four-factor model, and a second-order model in which feedback orientation was represented as a higher-order construct underlying the four first-order factors. Since multivariate normality was not confirmed by Mardia’s test, the robust maximum likelihood estimator methods were employed. Model adequacy was evaluated using multiple indices (CFI, TLI, RMSEA, SRMR), following conventional thresholds (Hu & Bentler, 1999).

Beyond factorial validity, internal consistency was assessed through Cronbach’s alpha and McDonald’s omega to capture both classical and model-based reliability. Convergent and discriminant validity were examined using Average Variance Extracted (AVE), Composite Reliability (CR), and the Heterotrait–Monotrait ratio (HTMT), which provide stronger evidence than item–total correlations alone. Criterion-related validity was tested by correlating FOS scores with theoretically relevant outcomes, namely job satisfaction (Bowling & Hammond, 2008) and perceptions of the feedback environment (Steelman & Levy, 2004), in line with meta-analytic findings consistently linking feedback orientation to these constructs.

Finally, measurement invariance was assessed on the same sample used for the CFA, across age, gender, educational level, and job role. To our knowledge, this is the first study to examine measurement invariance for the Italian adaptation of the FOS, and one of the first attempts to investigate cross-group comparability of the scale more broadly. This step was undertaken not to test specific hypotheses about group differences, but to ensure the psychometric robustness of the instrument across relevant subgroups. Establishing invariance is essential to distinguish true variance in the construct from variance attributable to measurement artifacts, thereby determining the conditions under which structural relations and group comparisons can be meaningfully interpreted (Cheung & Rensvold, 2002; Putnick & Bornstein, 2016).

The selected grouping variables reflect both standard demographic criteria and theoretically relevant dimensions. Gender is frequently included in validation studies to examine the equivalence of measurement models across groups, even in the absence of specific hypotheses (e.g., Dahling et al., 2010). Age was examined because systematic age-related differences in feedback orientation have been documented and are interpreted as substantive variation, reflecting developmental and motivational changes rather than measurement bias (Wang et al., 2015). Educational level was considered because, although previous research does not suggest a direct link between formal education and feedback orientation, it points to related constructs—such as learning goal orientation, cognitive engagement, and self-efficacy—that may vary with educational trajectories and influence feedback-related dispositions (Leenknecht et al., 2019; Anseel et al., 2013).

Finally, job role (distinguishing between more executive and more managerial positions) was included because it represents a structurally meaningful distinction within organizational contexts, typically associated with differences in responsibilities, autonomy, and exposure to feedback dynamics. Although no prior studies have specifically examined the measurement invariance of the FOS across job roles, testing invariance along this dimension offers an exploratory but theoretically grounded assessment of the generalizability of the scale across hierarchical levels, which is valuable for both future research and applied organizational settings.

## 3. Results

### 3.1. Exploratory Factor Analysis

Sampling adequacy was excellent (KMO = 0.93), indicating that the data were suitable for factor analysis. Bartlett’s test of sphericity was also highly significant (χ^2^(496) = 12,555.65, *p* < 0.001), confirming that the correlation matrix was factorable.

Initially, parallel analysis on the selected items supported a four-factor solution, which was extracted in EFA using PAF with oblimin rotation. The results indicated that most items loaded strongly on their intended factors (e.g., loadings > 0.70, see Table 2), with no problematic cross-loadings, and each item saturating primarily on its intended factor. The few lower loadings remained acceptable for exploratory work (e.g., item 8 = 0.393) (Costello & Osborne, 2005; Tabachnick & Fidell, 2013). Two items (Items 5 and 7) showed small cross-loadings, with secondary loadings of 0.30 on the Utility factor while still loading primarily on Accountability. Following Tabachnick and Fidell (2013), we considered loadings ≥ 0.32 as meaningful, and we inspected potential cross-loadings according to widely used recommendations (Costello & Osborne, 2005; Worthington & Whittaker, 2006). In line with these guidelines, although the secondary loadings reached the 0.30 level, the primary loadings remained higher, and the primary–secondary differences were slightly below the 0.20 threshold, indicating borderline item ambiguity rather than substantial cross-loading. No other item exceeded the 0.30 cutoff used to screen for potential cross-loadings. The eigenvalue analysis strongly supported the presence of four factors corresponding to the theoretical dimensions of the Feedback Orientation Scale. Specifically, the factor Utility showed the highest eigenvalue (2.66), followed by Social Awareness (2.31), Feedback Self-Efficacy (2.22), and Accountability (1.83). All four eigenvalues were above the conventional Kaiser criterion (eigenvalue > 1), indicating that each factor explains more variance than a single standardized item. Together, these four dimensions accounted for 56.4% of the total variance, a level consistent with recommended thresholds for multidimensional psychological scales. Factor intercorrelations were moderate to high (0.23–0.56), supporting the appropriateness of an oblique rotation. Overall, the EFA results provided empirical support for a coherent four-factor structure, consistent with the theoretical multidimensionality of feedback orientation.

### 3.2. Confirmatory Factor Analysis

Consistent with prior validations of the FOS (e.g., Linderbaum & Levy, 2010; Lilford et al., 2014), we compared alternative models to evaluate the latent structure. A one-factor model showed poor fit, whereas a correlated four-factor model substantially improved fit.

Among the tested solutions, the second-order model with four first-order factors loading on a global feedback orientation construct and including the correlated residuals not only provided the best overall fit, but was also the most consistent with the theoretical conceptualization of feedback orientation as a multidimensional yet integrative construct (Table 3).

In line with recent validation efforts (e.g., Fuentes-Cimma et al., 2025, Figure 2, p. 41), we introduced one correlated residual in the CFA model (specifically between items 1–2) to improve overall model fit, as suggested by modification indices exceeding 50. This decision was grounded not only in statistical criteria, but also in semantic and structural considerations. In both cases, the items appeared consecutively in the questionnaire and loaded on the same latent factor—Utility—which may have contributed to additional residual covariance beyond the target construct. Moreover, while not redundant, the items of the pair share partially overlapping content. Item 1 (“I find that feedback is critical for reaching my goals”) and item 2 (“Feedback is critical for improving performance”) both highlight the instrumental function of feedback in achieving professional objectives (see Table 1). To clarify whether the correlated residuals between Items 1 and 2 reflected true redundancy or merely shared substantive content, we estimated two additional CFA models in which either Item 1 or Item 2 was removed from the Utility factor. This step allowed us to compare the effectiveness of item deletion versus modeling the covariance directly. Full comparison results are reported in Table 4.

As expected, both reduced models yielded small improvements in global fit indices relative to the second-order specification without correlated residuals. However, these changes were marginal and did not translate into meaningful gains in internal consistency (α) or convergent validity (AVE). Across all specifications, the Utility factor remained psychometrically stable, and neither item demonstrated a uniquely problematic pattern of loadings or reliability. Thus, item removal did not offer substantive advantages in terms of scale performance.

Importantly, Items 1 and 2, while partially overlapping, capture complementary aspects of the instrumental value of feedback—progress toward goals and performance improvement—representing two facets that are both theoretically relevant. Eliminating either item would therefore narrow the conceptual coverage of the Utility dimension without improving the psychometric quality of the scale. Furthermore, in their cross-cultural adaptation, Fuentes-Cimma et al. (2025) also modeled residual covariance between these same items, arguing that their highly similar semantic content and adjacent positioning in the questionnaire may generate shared variance not fully captured by the latent factor.

For these reasons, we retained the full item set and modeled the residual covariance between Items 1 and 2. This approach provided a modest but consistent improvement in model fit while preserving the conceptual integrity and breadth of the construct.

Analyses of convergent and discriminant validity indicated that the Accountability dimension was the least robust component of the scale. Its AVE was 0.41, falling below the recommended 0.50 threshold (Kline, 2011; Brown, 2015; Fornell & Larcker, 1981), and its internal consistency was only moderate (α = 0.732). The HTMT between Utility and Accountability reached 0.89, exceeding the conservative 0.85 cutoff and approaching the 0.90 boundary proposed in recent SEM literature (Henseler et al., 2015; Hair et al., 2022), suggesting substantial conceptual overlap between the two factors. This strong association is theoretically plausible: perceiving feedback as important for achieving goals (Utility) naturally overlaps with perceiving a responsibility to act on feedback (Accountability). Although conceptually related, the two constructs remain distinguishable, with Utility reflecting the instrumental value of feedback, while Accountability captures its normative and duty-oriented component. All other HTMT values were well below the recommended thresholds, indicating adequate discriminant validity for the remaining factor pairs. To address the concern that Utility and Accountability might not represent empirically distinct constructs, we estimated an alternative second-order model in which all items from these two dimensions were combined into a single first-order factor. This three-factor model (Utility/Accountability, Social Awareness, Feedback Self-Efficacy) showed clearly poorer global fit than the four-factor solution (e.g., robust CFI ≈ 0.89, robust TLI ≈ 0.87 robust RMSEA ≈ 0.10, SRMR = 0.06), indicating that collapsing the two dimensions does not provide an adequate representation of the data. Overall, the results indicate that although perceiving feedback as useful may incline individuals to feel responsible for acting on it—a theoretically coherent association—the two dimensions remain empirically separable and functionally distinct within the measurement model.

Given the comparatively weaker convergent validity, reduced internal coherence, and the highest inter-factor overlap of the Accountability dimension, we proceeded to examine this factor through item-level diagnostics to identify potential sources of misfit.

Item-level diagnostics confirmed this pattern: Item 8 displayed the lowest corrected item–total correlation (r.drop = 0.45) and the lowest communality (h^2^ = 0.24), markedly weaker than the values observed for the remaining items (r.drop = 0.55–0.60; h^2^ = 0.51–0.69). To evaluate its impact on the model, we estimated a CFA excluding Item 8. The revised model showed a clear improvement in global fit (robust CFI increasing from 0.921 to 0.930; robust TLI from 0.903 to 0.913) and enhanced convergent validity, with the AVE for Accountability rising from 0.41 to 0.50. Discriminant validity also benefited from the removal, as the HTMT between Utility and Accountability decreased from 0.89 to 0.863, falling below the critical threshold. Importantly, modification indices no longer suggested local misfit within the Accountability factor once Item 8 was removed; the only residual correlation that remained necessary and theoretically justified concerned Items 1 and 2. Overall, the convergence of statistical and conceptual evidence indicated that Item 8 contributed disproportionately to the reduced coherence of the Accountability dimension. Its exclusion resulted in a more stable, internally consistent, and discriminant factor structure, and the item was therefore removed from the final validated version of the scale.

Importantly, the removal of Item 8 did not negatively affect the other dimensions: their reliability and convergent validity indices remained essentially unchanged, with differences never exceeding 0.001 across models. Utility retained an AVE of 0.597, Social Awareness an AVE of 0.526, and Feedback Self-Efficacy an AVE of 0.501, mirroring the values obtained in the initial model that included all items. This stability indicates that the exclusion of Item 8 strengthened the Accountability factor without altering the psychometric performance of the remaining dimensions. Overall, eliminating item 8 strengthened the coherence of the Accountability factor and reduced its redundancy with Utility, enhancing both convergent and discriminant validity without compromising the structure of the scale. Following the removal of Item 8, the Self-Efficacy factor remained the dimension with the weakest convergent validity, with an AVE of 0.495—acceptable but still indicative of limited shared variance among its indicators. Item-level diagnostics suggested that Item 16 was the primary source of this weakness. Modification indices revealed a large residual correlation between Items 15 and 16 (MI = 77.54), implying substantial redundancy beyond what is explained by the latent factor. This redundancy was theoretically plausible: the English wording of the two items confirms their semantic proximity, with Item 15 (“I feel confident when responding to both positive and negative feedback”) and Item 16 (“I feel self-assured when dealing with feedback”) both capturing a broad, overlapping sense of confidence in handling evaluative information rather than distinct aspects of feedback-related self-efficacy. Statistical evidence also supported the removal of Item 16. It showed the highest residual variance among all indicators of the factor (residual = 0.396; R^2^ = 0.403), suggesting limited contribution to the latent construct. We systematically compared alternative specifications—retaining both items with a correlated residual, removing Item 15 instead of Item 16, and removing Item 16 alone. Introducing the residual correlation between Items 15 and 16 failed to strengthen the factor, lowering the AVE to 0.466, while removing Item 15 produced negligible improvements. In contrast, removing Item 16 resulted in a clearer and more coherent Self-Efficacy factor, raising its AVE to 0.514 without negatively affecting reliability or the performance of the remaining dimensions. Taken together, these conceptual and statistical considerations indicated that Item 16 added redundancy rather than substantive information to the Self-Efficacy construct; its removal therefore produced a more parsimonious and psychometrically robust factor structure.

After the iterative refinement of the scale, including the removal of Items 8 and 16, the final model displayed a clear and well-defined hierarchical structure, with the four first-order dimensions loading onto a second-order Feedback Orientation factor (FOSV). The specification also retained a theoretically justified correlated residual between Items 1 and 2, reflecting their shared wording and closely aligned content. The overall model showed solid global fit (robust CFI = 0.942, robust TLI = 0.926, RMSEA = 0.057, SRMR = 0.052), indicating that the second-order structure captured the latent construct with good parsimony and minimal local misfit. Convergent validity was satisfactory, with AVE values of 0.597 for Utility, 0.500 for Accountability, 0.526 for Social Awareness, and 0.514 for Self-Efficacy, all at or above the recommended benchmark. Composite reliability coefficients were likewise adequate (0.821, 0.742, 0.816, and 0.752, respectively), confirming good internal consistency across dimensions. Discriminant validity was supported by the pattern of HTMT correlations, all below conventional cutoffs: the highest value, 0.838 between Utility and Accountability, remained within acceptable limits, while all other associations were considerably lower. Overall, once the residual dependence between Items 1 and 2 was accounted for and the item-level refinements were applied, the second-order model demonstrated a coherent factorial structure, balanced reliability, and solid convergent and discriminant validity, supporting the adequacy of the finalized measurement model.

Criterion-related (predictive) validity was supported: feedback orientation showed a 0.298 positive association with job satisfaction, consistent with meta-analytic evidence (rc ≈ 0.33; Katz et al., 2023); in our data, r = 0.25, *p* < 0.001. Construct validity was further corroborated 300 by the positive correlation between feedback orientation and the feedback environment, 301 consistent with meta-analytic evidence from the feedback environment meta-analysis (rc 302 ≈ 0.42; Katz et al., 2021) and discussed in the FO meta-analysis (Katz et al., 2023); in our data, r = 0.41, *p* < 0.001. This approach mirrors Linderbaum and Levy’s (2010) 304 original validation strategy, reinforcing the argument that the scale captures a dispositional tendency embedded within established feedback contexts.

After establishing the final second-order CFA structure, we tested its measurement invariance across key demographic and occupational subgroups. Invariance testing followed established recommendations for multi-group CFA (Cheung & Rensvold, 2002; Chen, 2007; Putnick & Bornstein, 2016). We sequentially evaluated configural, metric, scalar, and strict invariance across four grouping variables: gender (male and female; participants identifying with other gender categories were excluded from this analysis due to the very small subgroup size, *n* = 8), age (≤40 vs. >40 years), educational level (lower vs. higher education), and job role (managerial vs. non-managerial positions).

Model comparisons were evaluated using changes in CFI, SRMR, and RMSEA. In line with established recommendations (Cheung & Rensvold, 2002; Chen, 2007), measurement invariance was considered tenable when ΔCFI ≤ 0.010, ΔSRMR ≤ 0.010 for scalar/strict invariance and ≤0.030 for metric invariance, and ΔRMSEA ≤ 0.015. Robust (Yuan–Bentler) fit indices were used for all evaluations.

Table 5 reports the complete fit indices and Δ values for each step.

We acknowledge that the RMSEA of the configural model lies in the 0.08–0.09 range. Following the interpretative guidelines proposed by MacCallum et al. (1996), RMSEA values between 0.08 and 0.10 reflect a mediocre level of fit, which does not necessarily indicate poor model performance. This interpretation is consistent with the observation that RMSEA tends to be more severe in complex, multifactor item-level models. As noted by Marsh et al. (2004), such models frequently yield higher RMSEA values due to their structural complexity, even when the underlying factor solution is reasonable. Chen (2007), in particular, demonstrated that RMSEA can fluctuate with model complexity and degrees of freedom, sometimes producing indications of non-invariance even when constraints are tenable. At the same time, we recognize that the mediocre RMSEA of the configural model calls for a cautious interpretation. As this is the first study to examine measurement invariance for the Italian adaptation of the FOS, we consider the present findings supportive but not definitive. Further research is needed to determine whether the observed RMSEA values reflect methodological characteristics of the index in complex models or subtle group differences that warrant additional investigation.

Gender: The configural model showed acceptable CFI (0.914) and SRMR (0.057), together with an RMSEA of 0.089, which falls within the *mediocre but acceptable* range for complex multifactor models (MacCallum et al., 1996). This suggests that men and women share a broadly similar factor structure, although some misfit is present and should be interpreted cautiously.

Metric invariance was supported (ΔCFI = –0.001; ΔSRMR = 0.004), indicating that factor loadings are comparable across genders. Scalar invariance was also broadly supported (ΔCFI = –0.004; ΔSRMR = 0.001), suggesting similar item intercepts, although again the RMSEA remains modestly elevated. Strict invariance showed a more notable drop in CFI (–0.013), indicating only partial support for equality of residual variances. The factor structure and loadings appear stable across gender, and latent mean comparisons are tentatively possible, but comparisons of raw scores are not recommended, and all interpretations should remain cautious given the mediocre RMSEA and the decrease in CFI at the strict level.

Age: The configural model displayed CFI = 0.918 and SRMR = 0.056, with an RMSEA of 0.088, again indicating mediocre but acceptable fit. This suggests that the basic factor structure is reasonably consistent across age groups.

Metric and scalar invariance showed stable ΔCFI values (–0.005 for both) and acceptable ΔSRMR values (0.009 and 0.002), supporting comparable loadings and intercepts across younger and older workers. At the strict level, ΔCFI reached –0.026, indicating that error variances differ more substantially. Across age groups, structure, loadings, and intercepts appear broadly comparable, but strict invariance is not supported. Latent means may be compared with caution; raw-score comparisons should be avoided.

Education: The configural model (CFI = 0.913; SRMR = 0.053; RMSEA = 0.089) again showed mediocre but acceptable fit, supporting a similar factor structure across education levels. Metric and scalar invariance were supported (ΔCFI ≤ −0.001; ΔSRMR ≤ 0.004), indicating stability of loadings and intercepts. Strict invariance also showed acceptable ΔCFI (–0.010) and ΔSRMR (0.002). The measurement model is largely invariant across education groups, enabling cautious comparisons of latent means. As in other groups, the mediocre RMSEA of the configural model suggests interpreting results with restraint.

Job role: The configural model showed acceptable CFI (0.912) and SRMR (0.056), with an RMSEA of 0.091. This aligns with the “mediocre but acceptable” range typical for complex item-level models.

Metric invariance was supported (ΔCFI = −0.004; ΔSRMR = 0.006). Scalar invariance also met the recommended criteria (ΔCFI = −0.003; ΔSRMR = 0.001). Strict invariance yielded ΔCFI = −0.003 and a negligible ΔSRMR difference, indicating a stable model across constraints. This is the strongest invariance pattern among the groups, within the limits of the mediocre RMSEA observed at the configural level. The factor structure, loadings, and intercepts are comparable across managerial and non-managerial employees, and error variances do not show substantial differences. Latent means can be compared, though the mediocre RMSEA of the configural model still suggests cautious interpretation.

## 4. Discussion

The primary aim of this study was to validate the FOS in the Italian context, thereby contributing to the cross-cultural generalizability of the construct and providing a reliable instrument for applied research and practice. Guided by the theoretical model of Linderbaum and Levy (2010), we tested the factorial structure, reliability, and multiple forms of validity—convergent, discriminant, and criterion-related—of the scale.

Consistent with the theoretical model proposed by Linderbaum and Levy (2010), both exploratory and confirmatory factor analyses supported a second-order four-factor structure—Utility, Social Awareness, Accountability, and Feedback Self-Efficacy. This replicates results obtained in other cultural and occupational settings (e.g., Lilford et al., 2014; Fuentes-Cimma et al., 2025), reinforcing the conceptualization of feedback orientation as a multidimensional disposition that captures how individuals perceive, value, and respond to feedback. By recovering the expected structure, our findings offer tentative evidence that the construct may generalize to the Italian context, indicating that the FOS holds potential as an instrument for assessing feedback orientation in the national labor market.

In addition to confirming the second-order four-factor structure originally proposed by Linderbaum and Levy (2010), this study extends prior validations in several ways. First, whereas earlier studies primarily relied on EFA during scale development (Linderbaum & Levy, 2010) or CFA in applied contexts (Lilford et al., 2014; Braddy et al., 2013), we combined exploratory and confirmatory analyses—guided by parallel analysis and rigorous model comparisons—thus providing evidence for model selection. Second, we formally assessed convergent and discriminant validity using AVE and HTMT, offering a more stringent test than traditional approaches. Third, we examined the measurement invariance of the FOS.

Exploratory factor analysis provided strong initial support for the four-factor structure of the FOS, with all items loading on their intended factors and no problematic cross-loadings. The solution explained a substantial proportion of the variance and was consistent with the theoretical model proposed by Linderbaum and Levy (2010). Confirmatory factor analysis further corroborated this structure: the unidimensional model demonstrated poor fit, whereas both the correlated four-factor and the second-order models yielded substantially better indices. Among the tested solutions, the second-order model with four first-order factors and selected correlated residuals provided the most theoretically coherent and statistically adequate representation of feedback orientation as a multidimensional but integrative construct.

Convergent validity was supported for three of the four factors, whose AVE values exceeded the recommended 0.50 threshold, whereas the Accountability dimension displayed comparatively weaker convergence—an issue consistent with its lower internal consistency and item cohesion. Discriminant validity was generally adequate, although the association between Utility and Accountability was relatively high. This overlap likely reflects a substantive proximity between the two constructs in the Italian context—perceiving feedback as useful often aligns with feeling responsible for acting on it—yet their conceptual distinction remains theoretically meaningful and warrants further examination. Criterion-related validity was also supported, with FOS scores correlating in the expected directions with job satisfaction and the feedback environment, in line with meta-analytic evidence (Katz et al., 2023).

Taken together, these findings reflect a multi-step refinement process in which items were screened both qualitatively and quantitatively. As a result, the Italian adaptation consists of a concise 14-item version of the FOS. While this represents a reduced item set compared to the original scale, the retained items continue to map onto the four theoretical dimensions proposed by Linderbaum and Levy (2010), allowing the construct to be assessed in a manner that remains conceptually coherent and psychometrically adequate.

Importantly, this study represents the first systematic examination of measurement invariance for the FOS within the context of an Italian validation. Based on the criteria recognized as most robust for invariance testing—namely ΔCFI and ΔSRMR (Cheung & Rensvold, 2002; Chen, 2007)—our findings support the equivalence of the measurement structure across gender, age groups, job roles, and educational levels. At the same time, the behavior of RMSEA across increasingly constrained models introduces an element of caution: although RMSEA values remained within acceptable limits, their fluctuations likely reflect methodological sensitivity rather than substantive non-invariance. These results therefore provide initial but not definitive evidence of invariance, and further studies are needed to consolidate these findings in broader and more heterogeneous samples.

## 5. Limitations

Although this study provides evidence for the psychometric soundness of the FOS in the Italian context, several limitations should be acknowledged. First, the data were collected exclusively through self-report questionnaires, which are susceptible to biases such as social desirability and common method variance. Third, our evaluation of measurement invariance—the first conducted of the FOS—suggests general equivalence across demographic groups when relying on ΔCFI and ΔSRMR, the criteria considered most stable in invariance testing. However, RMSEA displayed fluctuations across increasingly constrained models. Although these values remained within acceptable limits and are likely attributable to RMSEA’s known sensitivity to model complexity, this pattern introduces uncertainty and warrants further investigation in future studies. Furthermore, the cross-sectional design prevents drawing conclusions about the stability or malleability of feedback orientation over time. Finally, the study relied on convenience and snowball sampling, which restricts the representativeness of the sample. Although respondents were recruited from multiple regions, participants from Southern Italy were underrepresented, reflecting the structure of the recruitment networks. Consequently, the findings should not be generalized to the entire Italian workforce. The sample is sufficiently heterogeneous for psychometric purposes, but caution is required when extrapolating results or comparing subgroups with uneven representation.

Future psychometric studies should pay particular attention to the Accountability facet. This dimension was affected by the removal of one item during the qualitative refinement and another during the quantitative analyses (item 8), which may have reduced its breadth of content. Additional work is therefore needed to ensure that the Accountability domain is fully represented—for example, by developing or testing alternative items that capture different expressions of responsibility in feedback processes, such as perceived expectations, personal standards, or contextual norms for acting on feedback. Moreover, to consolidate criterion evidence in applied settings, time-lagged designs (e.g., assessing FOS at T1 and job attitudes at T2) can offer a feasible step toward establishing temporal precedence. Furthermore, the FOS emerges as a useful diagnostic for profiling feedback readiness and tailoring development. Because feedback orientation is plausibly malleable, organizations can evaluate whether targeted interventions (e.g., feedback-literacy training, developmental framing and goal-setting routines, supervisor coaching) produce measurable changes in FOS scores over time, using pre–post–follow-up assessments and linking change to relevant outcomes such as job satisfaction and appraisal reactions. In conclusion, the Italian FOS emerges as a promising, though still preliminary, instrument for assessing feedback orientation that can both advance research across Italian organizational settings and guide evidence-informed interventions aimed at enhancing feedback readiness, appraisal reactions, and employee well-being.

## 6. Theoretical Implications and Practical Implications

The present study contributes to the literature on feedback orientation by providing an initial validation of the Italian FOS and by supporting the multidimensional structure proposed by Linderbaum and Levy (2010) and London and Smither (2002). During the adaptation phase, four items—one per dimension—were removed during the qualitative screening phase based on issues of ambiguity, redundancy, or conceptual misalignment.

Overall, the adaptation process improved semantic clarity and cultural appropriateness while preserving the theoretical coherence of the construct. Nevertheless, the removal of items prior to quantitative analyses implies that the Italian version represents a refined 14-item adaptation rather than a direct linguistic translation of the 20-item scale. This should be taken into account when considering the comparability of the Italian version with the original instrument and when planning future cross-cultural validation efforts.

By extending validation evidence beyond English-speaking contexts, this study broadens the cross-cultural examination of FO and provides an initial foundation for systematic research within the Italian labor market, where empirical evidence has been limited. The availability of an Italian version with acceptable psychometric properties supports future investigations into FO as both an antecedent and moderator of workplace behaviors, while acknowledging that further research is needed to strengthen certain subdimensions—particularly Accountability—and to confirm measurement stability across groups (Dahling et al., 2010; Gabriel et al., 2014). Methodologically, this study advances prior work by combining exploratory and confirmatory approaches, evaluating convergent and discriminant validity with established criteria (Worthington & Whittaker, 2006; Brown, 2015), and conducting a formal examination of measurement invariance. Taken together, these contributions provide a foundation for continued theoretical and empirical developments on feedback processes, performance management, and career sustainability in the Italian context.

From a practical standpoint, the Italian FOS represents a promising tool for assessing employees’ receptivity to feedback, while recognizing that some subdimensions may benefit from future refinement. The scale can support interventions aimed at strengthening feedback literacy and self-efficacy in using feedback, and it may be useful for monitoring developmental changes over time. By differentiating levels of feedback orientation, HR professionals and managers can design tailored interventions—such as feedback-literacy programs, developmental framing of appraisal processes, or coaching initiatives—that strengthen self-efficacy in using feedback and support sustainable employability. Because FO is malleable, the scale can also be used to monitor changes over time and evaluate the effectiveness of organizational practices aimed at fostering feedback readiness. Importantly, although meta-analytic evidence confirms the central role of FO in shaping feedback processes and outcomes (Katz et al., 2021), there is still a lack of experimental studies that directly manipulate FO in organizational contexts. The validated Italian FOS thus provides a valuable foundation for both monitoring FO and informing future interventions that seek to actively develop employees’ feedback receptivity. Ultimately, applying the FOS in organizational contexts can inform evidence-based HRM practices that promote individual well-being, career sustainability, and long-term organizational effectiveness.

## Figures and Tables

**Table 1:** Expert evaluation across qualitative criteria.

Item	Semantic Clarity	Contextual Relevance	Ambiguity/Multiple Interpretations
“Feedback contributes to my success at work”	The term *“success”* was judged as vague and semantically undefined.	Experts noted that the idea that feedback contributes to work-related success is generally compatible with workplace dynamics. However, the wording remains overly generic and culturally loaded: the notion of “success” carries different meanings across professions, organizational cultures, and career orientations. As a result, the item’s relevance cannot be consistently interpreted, despite its broad alignment with workplace themes.	Not formally ambiguous, but open to widely diverging subjective interpretations.
“I feel obligated to make changes based on feedback”	Linguistically clear but conceptually heavy.	The notion of “obligation” does not reflect typical feedback dynamics, where feedback is more often framed as guidance, suggestions for improvement, or normative expectations rather than explicit mandates to change.	Highly ambiguous: may imply ethical duty, social pressure, or coercion.
“I rely on feedback to help me make a good impression”	Understandable but perceived more as impression management rather than social awareness	Experts noted that the item blends social-awareness processes with self-promotion motives. Since Social Awareness concerns understanding how one is perceived, whereas “making a good impression” reflects strategic self-presentation, the relevance of this phrasing to the intended construct was considered weak.	No specific ambiguity, but the focus on image may lead to overly subjective interpretations.
“Compared to others, I am more competent at handling feedback”	Clear wording but perceived as forced.	Experts indicated that comparing oneself to others in handling feedback is atypical but not entirely implausible in some work settings. However, the item stands out conceptually: while other items in the scale capture self-referential, introspective beliefs about one’s own feedback orientation, this item introduces a comparative judgment that relies on assumptions about others’ competencies. This shift in evaluative focus makes its contextual relevance only moderate and its conceptual fit with the subscale weaker.	Moderate ambiguity: unclear how an individual would assess being “more competent than others.”

**Table 2:** Exploratory factor analysis results FOS.

Item	Utility	Social-Awareness	Accountability	Feedback Self-Efficacy
Item 1	**0.760**	0.047	0.032	0.000
Item 2	**0.940**	−0.011	−0.062	−0.010
Item 3	**0.591**	0.061	0.175	0.104
Item 4	**0.443**	0.104	0.290	0.091
Item 5	0.308	0.031	**0.462**	0.033
Item 6	−0.038	0.025	**0.844**	−0.003
Item 7	0.308	−0.011	**0.449**	0.118
Item 8	−0.024	0.162	**0.393**	0.053
Item 9	0.132	**0.599**	0.135	0.036
Item 10	−0.051	**0.776**	0.092	−0.004
Item 11	−0.013	**0.892**	−0.086	−0.034
Item 12	0.064	**0.574**	−0.034	0.112
Item 13	−0.032	0.023	−0.072	**0.799**
Item 14	0.134	0.023	0.038	**0.659**
Item 15	−0.100	−0.042	0.042	**0.759**
Item 16	0.077	0.007	−0.006	**0.610**

Note: FOS = Feedback Orientation Scale. Bold values indicate the primary factor loadings for each item.

**Table 3:** Confirmatory Factor Analysis Model comparison.

Model	CFI	TLI	RMSEA	SRMR
One-factor	0.697	0.651	0.147	0.101
Four-factor	0.892	0.868	0.091	0.066
Second-order (no correlated residuals)	0.892	0.871	0.090	0.067
Four-factor correlated residuals	0.921	0.901	0.078	0.058
Second-order correlated residuals	0.921	0.903	0.077	0.058

Note: CFI = Comparative Fit Index; TLI = Tucker–Lewis Index; RMSEA = Root Mean Square Error of Approximation; SRMR = Standardized Root Mean Square Residual.

**Table 4:** Alternative CFA Models Testing Item Removal (Second-order Structure).

Model	CFI	TLI	RMSEA	SRMR	α UT	α AC	α SA	α SE	AVE UT	AVE AC	AVE SA	AVE SE
A	0.892	0.871	0.090	0.067	0.893	0.735	0.798	0.793	0.664	0.415	0.501	0.503
B	0.908	0.885	0.087	0.060	0.839	0.731	0.797	0.792	0.633	0.414	0.500	0.502
C	0.912	0.891	0.084	0.059	0.815	0.732	0.797	0.793	0.599	0.414	0.501	0.503

Note: *Model A* = Full model with no correlated residuals, *Model B* = Without Item 1, *Model C* = Without Item 2.

**Table 5:** Measurement invariance of the final second-order FOS model.

Group	Model	CFI	RMSEA	SRMR	ΔCFI	ΔRMSEA	ΔSRMR
Gender	Configural	0.914	0.089	0.057	—	—	—
	Metric	0.913	0.088	0.061	−0.001	−0.002	0.004
	Scalar	0.909	0.087	0.062	−0.004	−0.000	0.001
	Strict	0.896	0.090	0.064	−0.013	0.002	0.002
Age	Configural	0.918	0.088	0.056	—	—	—
	Metric	0.914	0.087	0.066	−0.005	−0.001	0.009
	Scalar	0.908	0.087	0.067	−0.005	0.000	0.002
	Strict	0.882	0.095	0.071	−0.026	0.008	0.004
Job Role	Configural	0.912	0.091	0.056	—	—	—
	Metric	0.908	0.090	0.061	−0.004	−0.001	0.006
	Scalar	0.905	0.089	0.063	−0.003	−0.001	0.001
	Strict	0.902	0.087	0.062	−0.003	−0.002	−0.000
Education	Configural	0.913	0.089	0.053	—	—	—
	Metric	0.911	0.087	0.058	−0.001	−0.002	0.004
	Scalar	0.911	0.085	0.058	−0.001	−0.002	0.000
	Strict	0.900	0.086	0.060	−0.010	0.001	0.002

*Note.* CFI = Comparative Fit Index; TLI = Tucker–Lewis Index; RMSEA = Root Mean Square Error of Approximation; SRMR = Standardized Root Mean Square Residual. ΔCFI, ΔRMSEA, and ΔSRMR represent the change in model fit relative to the immediately less constrained model (Metric vs. Configural, Scalar vs. Metric, Strict vs. Scalar).

## Data Availability

The data presented in this study are available on request from the corresponding author.

## Abbreviations

The following abbreviations are used in this manuscript:

FOS	Feedback Orientation Scale
FO	Feedback Orientation
CFA	Confirmatory Factor Analyses
EFA	Exploratory Factor Analyses
PAF	Principal Axis Factoring
HTMT	Heterotrait–Monotrait ratio
AVE	Average Variance Extracted
CR	Composite Reliability
CFI	Comparative Fit Index
TLI	Tucker–Lewis Index
RMSEA	Root Mean Square Error of Approximation
SEM	Structural Equation Modeling
SDGs	Sustainable Development Goals
SRMR	Standardized Root Mean Square Residual

## Appendix A. Italian Version of the Feedback Orientation Scale (FOS)

The Italian version of the FOS consists of 14 items across four dimensions: Utility, Responsibility, Social Awareness, and Feedback Self-Efficacy (Linderbaum & Levy, 2010). Items were translated and adapted using a back-translation and expert review procedure, following international guidelines. Responses are provided on a 5-point Likert scale (1 = Strongly disagree; 5 = Strongly agree).

**Dimensions**	**N.**	**Items ITA**	**Items ENG**
**Utility**	1	Trovo che i feedback siano fondamentali per raggiungere i miei obiettivi	I find that feedback is critical for reaching my goals
	2	I feedback sono fondamentali per migliorare le mie prestazioni	Feedback is critical for improving performance.
	3	Prendo in considerazione i feedback ricevuti, per sviluppare le mie competenze sul lavoro	To develop my skills at work, I rely on feedback.
	4	I feedback dei responsabili possono aiutarmi a progredire in un’organizzazione	Feedback from supervisors can help me advance in a company.
**Accountability**	5	Sento la responsabilità di dar seguito ai feedback in modo appropriato	I hold myself accountable to respond to feedback appropriately.
	6	Se il mio responsabile mi dà dei feedback, è mio dovere applicarli nel mio lavoro	If my supervisor gives me feedback, it is my responsibility to respond to it.
	7	È mia responsabilità sfruttare al meglio i suggerimenti ricevuti durante i feedback per migliorare la mia performance	It is my responsibility to apply feedback to improve my performance.
	8	Non mi sento appagato fino a quando non ho messo in atto i suggerimenti ricevuti nei feedback	I don’t feel a sense of closure until I respond to feedback.
**Social Awareness**	9	Prendendo in considerazione i feedback, sono più consapevole di ciò che gli altri pensano su di me	Using feedback, I am more aware of what people think of me.
	10	I feedback mi aiutano a gestire l’impressione che faccio sugli altri	Feedback helps me manage the impression I make on others.
	11	I feedback mi fanno capire come vengo percepito dagli altri	Feedback lets me know how I am perceived by others.
	12	Cerco di essere consapevole di ciò che gli altri pensano su di me	I try to be aware of what other people think of me.
**Feedback Self-Efficacy**	13	So di poter essere in grado di gestire i feedback che ricevo	I know that I can handle the feedback that I receive.
	14	Credo di avere la capacità di utilizzare i feedback ricevuti in modo efficace	I believe that I have the ability to deal with feedback effectively.
	15	Mi sento sicuro di me quando si tratta di ricevere dei feedback sia positivi che negativi	I feel confident when responding to both positive and negative feedback.
	16	Mi sento a mio agio quando mi vengono dati dei feedback	I feel self-assured when dealing with feedback.
